# Transformation of Canine Lymphoma/Leukemia to More Aggressive Diseases: Anecdotes or Reality?

**DOI:** 10.3389/fvets.2015.00042

**Published:** 2015-10-07

**Authors:** Stefano Comazzi, Luca Aresu, Laura Marconato

**Affiliations:** ^1^Department of Veterinary Sciences and Public Health (DIVET), University of Milan, Milan, Italy; ^2^Department of Comparative Medicine and Food Science, University of Padua, Padua, Italy; ^3^Centro Oncologico Veterinario, Sasso Marconi, Italy

**Keywords:** dog, lymphoma, leukemia, transformation, indolent, Richter syndrome, blast crisis, DLBCL

## Abstract

Transformation is the evolution of an indolent lymphoma/leukemia to an aggressive lymphoma, typically harboring a very poor prognosis. This phenomenon is well described in humans, but underestimated in dogs although recognized as a possible evolution of indolent lymphomas/leukemias. In canine chronic leukemias, blast crisis (mainly in myeloid) and Richter syndrome (transformation into a high grade lymphoma) (mainly in B-cell lymphocytic leukemia) have been reported. Transformation is a possible event also in canine low grade lymphomas, although rare. The increased knowledge has also generated new questions and posed challenges that need to be addressed to improve outcome, including the recognition of the clinical characteristics at diagnosis associated with a higher risk of transformation in an attempt of anticipating the typical evolution.

## Introduction

Transformation of an indolent low grade lymphoma into a more aggressive histotype is a well-known process in human hemato-oncology, generally linked to unfavorable prognosis and poor outcome. Histological transformation of follicular lymphoma (FL) into more aggressive lymphoma subtypes is a pivotal event in the natural history of patients, representing one of the major challenges in the management of patients with FL. Chronic lymphocytic leukemia (CLL) are commonly reported to progress into aggressive high grade lymphoma (Richter syndrome, RS) or acute leukemia (blast crisis). Transformation has been reported also in other subtypes of low grade lymphomas, either B-cell (marginal zone lymphoma – MZL, mantle cell lymphoma – MCL, mucosa-associated lymphoid tissue lymphoma – MALT) or T-cell in origin (mycosis fungoides – MF), and generally implies the histological effacement of the nodal architecture and/or the increase in the proportion of large cells (1), together with the onset of clinical signs.

Even though the term “transformation” implies the direct evolution of neoplastic cells into more aggressive ones this might not be the case and efforts have been made to determine the origin of transformed neoplastic cells (arising from the same clones or from different ones) as well as to identify possible pathogenetic mechanisms driving transformation. As a matter of fact, transformation implies the shift to a more aggressive clinical behavior and justifies the need of an aggressive therapeutic approach.

In veterinary oncology, the issue of transformation of lymphoma/leukemia into more aggressive forms is debated but anecdotic.

Aim of the present article is to review the veterinary literature and to describe the authors’ experience.

## Basis of Transformation

Different factors, including genetic modifications, epigenetic transformation, and microenvironment, may contribute to reprograming the original neoplastic cells and lead to transformation into a more aggressive histological pattern.

During transformation, two different scenarios may occur: (1) a specific neoplastic clone undergoes multiple and sequential mutations, leading to less differentiated neoplastic cells with a more aggressive behavior or (2) different clones from the original neoplastic ones desegregate and proliferate, thereby replacing the previous tumor (2).

Both the scenarios may be alternatively possible. In most cases of RS, neoplastic cells are clonally related to the original CLL, whereas in a minority of cases (<20%), new clones develop as a “*de novo*” neoplasm (3). However, even if clonally related, a direct evolution of indolent into the new aggressive neoplastic cells is not universally accepted. For instance, there is some evidence that the transformed tumor may arise from common precursor cells rather than directly evolving from the primary clone, as described for FL (2).

## Hematopoietic Neoplasm Undergoing Transformation

### Chronic myeloid leukemia

If untreated, human chronic myeloid leukemia (hCML) may typically transform into aggressive leukemia. hCML may evolve to an accelerated phase characterized by increased percentages of abnormal blast cells (10–19% of total nucleated cells in the bone marrow), unresponsive thrombocytopenia/thrombocytosis, basophilia, leukocytosis, unresponsive anemia, and splenomegaly (4). The final evolution of this accelerated phase is the blast crisis, in which the blast percentage in the bone marrow exceeds 20%, and clinical symptoms become apparent. Most hCML in blast crisis resemble acute myeloid leukemia (AML), whereas approximately one-third of them mimic acute lymphoid leukemia (ALL) (5). Based on the above, it may be speculated that transformed neoplastic cells likely derive from a common undifferentiated precursor rather than directly from hCML neoplastic cells. The immunophenotype of transformed cells reveals an undifferentiated pattern, including positivity to CD34 and CD7 in many cases (6). Chromosomal and molecular secondary aberrancies, in addition to the classic Philadelphia chromosome with t(9:22), generally occur during transformation (7). These changes may reflect the accumulation of genetic abnormalities of neoplastic cells in addition to the primary BCR–ABL translocation, thus preventing cell differentiation and leading to uncontrolled proliferation (4).

Chronic myelomonocytic leukemia (CMML) is a myeloproliferative/myelodysplastic neoplasm that naturally evolves to AML in 15–30% of cases and only rarely to ALL. According to the percentage of blast cells, CMML are subdivided into two groups (type 1 and type 2 CMML), with type 2 roughly corresponding to the accelerated phase of CML (8).

#### Canine Chronic Myeloid Leukemia

**Figure 1:**
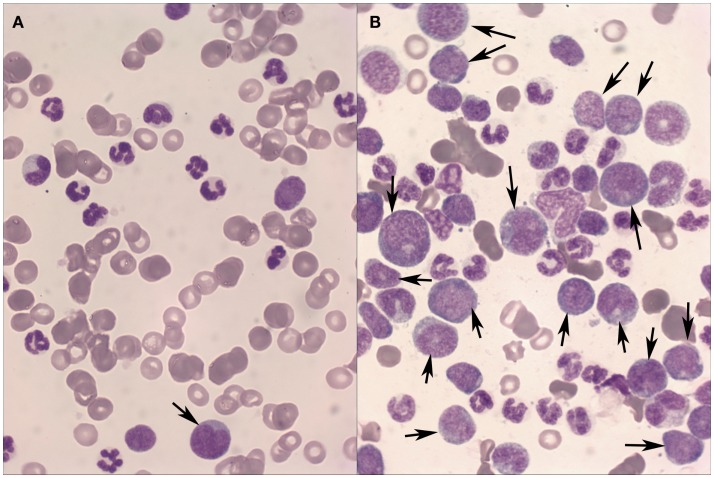
**Peripheral blood (A) and bone marrow (B) from a dog with Chronic myeloid leukemia in blast transformation**. A high percentage of blast cells (arrows) are found mainly in the bone marrow aspirate. May–Grünwald–Giemsa stain, 40×.

To the authors’ knowledge, large series on transformation of canine CML or CMML have not been described. However, some isolated cases of blast crisis have been reported (9), but it is generally considered as rare in dogs (10). This is probably due to the rarity of myeloproliferative neoplasms in dogs and to the difficulties in distinguishing between blast crisis and primary AML, if clinical and laboratory history is inadequate. However, in the authors’ experience, accelerated phase and blast crisis in dogs with CML and CMML are not exceptional findings (Figure 1). The percentage of canine myeloproliferative neoplasms undergoing blast transformation may be difficult to estimate, also because CML and CMML may arise asymptomatically, and clinical signs may become evident during transformation.

### Chronic lymphocytic leukemia

Chronic lymphocytic leukemia is a common hematological neoplasm both in humans and dogs. Human CLL (hCLL) have a characteristic B CD5^+^ phenotype. Three main different types of evolution of CLL have been reported (3): (1) RS, (2) prolymphocytic leukemia, and (3) blast crisis.

Richter syndrome refers to the rapid development of an aggressive lymphoma in patients with CLL, occurring in 2–20% of hCLL. Most frequently (90% of cases), B-CLL transforms into diffuse large B-cell lymphoma (DLBCL), rarely (10%) into Hodgkin lymphoma (11).

In the pathogenesis of RS, the large cells diffusely infiltrating the lymph node structure may arise from a transformation of the original CLL clone (approximately 80% of cases), due to the accumulation of genetic and/or epigenetic lesions. In the remaining 20% of cases, RS represents a “*de novo*” lymphoma and the pathogenesis is likely related to host genetic background or microenvironmental dysfunction, enhancing the probability of DLBCL (12). Some molecular markers have been associated with RS transformation. They include clinical (advanced Rai stage), immunophenotypic (ZAP-70, CD38, CD49d), genetic (del-17p, del-11q) characteristics at the time of CLL diagnosis, and single nucleotide somatic mutations, including TP53 disruption, c-myc activation, CDKN2A loss, and NOTCH1 mutations (3).

Prolymphocytic leukemia is uncommon and is characterized by increased cells at their intermediate development stage, massive splenomegaly, and onset of clinical signs. Prolymphocytes are medium sized cells with visible nucleoli and decondensed chromatin.

Finally, blast crisis may occur as described for CML cases, but it is usually rare.

#### Canine Chronic Lymphocytic Leukemia

In contrast with human counterpart, about two-thirds of canine CLL are of T phenotype. Data regarding evolution and possible transformation of canine chronic lymphocytic leukemia (cCLL) in more aggressive neoplasms are fragmentary. RS has been reported in 2 cases out of a series of 22 cCLL (13). Recently, we reported 8 cases of RS (6 having B-cell phenotype and 2 having T-cell phenotype) in a cohort of 153 cCLL cases (93 T-CLL, 55 B-CLL, 5 atypical CLL) (14). RS in B-cCLL showed a prevalence similar to that described in hCLL (10.9%). During Richter transformation, dogs exhibited a decrease of lymphocyte counts, often associated with anemia and thrombocytopenia, lymphadenomegaly with cytological and histological features of DLBCL, and onset of clinical signs. Outcome was poor, with a median survival time of 41 days. In agreement with the human literature, RS is also a possible evolution of cCLL, moreover in the case of B phenotype. However, clonality has been evaluated in two cases only, and it was not possible to determine if the described cases were all clonally related or represented a “*de novo*” tumor.

Regarding the other types of transformation, Takahashi et al. (15) reported blast crisis in one dog with T-LGL CLL with a shift of immunophenotype from CD3^+^CD8^+^ CLL to CD3^−^CD8^−^ ALL, coupled with anemia and thrombocytopenia. Once again, it was impossible to determine if the two diseases were clonally related. Other cases of blast crises have been reported in different case series (9). Burnett et al. (16) reported a case of B-cCLL evolving into a clonally related multiple myeloma following chemotherapy.

Transformation of cCLL into prolymphocytic leukemia is poorly reported but, to the authors’ experience, this evolution is likely underestimated also due to the lack of specific markers. Evolution to prolymphocytic leukemia should be suspected if the morphological evaluation of the blood smear reveals medium sized nucleolated cells, in addition to an aggressive clinical course.

### Follicular lymphoma

Follicular lymphoma is the most frequent human lymphoma and the transformation to a high grade lymphoma is a well-known evolution, ranging from 10 to 60% of cases (1). Typically, FC transforms into DLBCL, whereas transformation into Burkitt lymphoma, ALL, or lymphoblastic lymphoma seems to occur at a lesser incidence. An increase in the proportion of centroblasts and the parallel effacement of the follicular architecture are pivotal events, and transformed DLBCL frequently maintains the germinal center (GC) pattern expressing BCL6 and CD10 and the genetic features characteristic of FL, such as t(14:18). Recent studies suggested that transformation of FL into DLBCL is frequently a non-linear event, with transformed cells deriving more frequently from a common progenitor harboring driver mutations rather than directly from the predominant clone of FL (17). Several events may foster transformation, including genetic alterations (MYC, p53, p16, BCL6, BCL2), epigenetic factors, and the microenvironment (follicular dendritic cells, microvessel) and intra follicular non-neoplastic lymphoid cells (CD4^+^ and Tregs).

From a clinical point of view, the rise of B symptoms (fever, night sweats, and weight loss), nodal growth, hypercalcemia, and increased LDH level are common features. Most studies report a poor prognosis, with survival after transformation ranging from months to few years.

#### Canine Follicular Lymphoma

**Figure 2:**
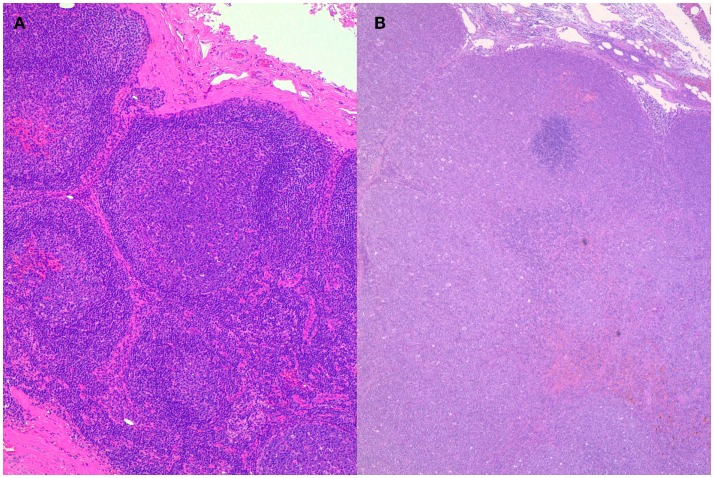
**Histopathology of lymph nodes from the same dog at presentation (A) and after transformation (B)**. A follicular lymphoma was diagnosed at presentation transforming into diffuse large B-cell lymphoma after therapy and recurrence. Hematoxylin–Eosin stain, 10×.

In contrast with humans, FLs are rare in dogs, representing no more than 1% of cases (18). To the authors’ knowledge, transformation of canine follicular lymphoma (cFL) in DLBCL has not been directly reported. This may be due to the poor propensity to have a histopathological diagnosis at presentation and moreover at relapse, rather than to a true low incidence. Between 2011 and 2015, at one of the authors’ institutions (LM), 13 dogs with cFL were diagnosed and treated. Data concerning signalment, symptoms, initial staging work-up, treatment, and follow-up were known in all cases. Histological diagnosis was obtained in all cases. First-line therapy for these 13 dogs was administered according to protocols used at that time and consisted of dose-intense chemotherapy. Dogs were fully restaged at progression, and all of them underwent lymphadenectomy and histopathological evaluation. The diagnosis of transformation was always based on histology and was defined by the histological features of DLBCL. Transformation was documented in 2 (15.4%) of 13 dogs at their first recurrence (Figure 2). Both dogs were treated with rescue chemotherapy and eventually died with progression after few months.

In a previous study by the group, Array Comparative Genomic Hybridization in canine DLBCL was carried out at diagnosis and at relapse to compare the profiles (19). Overall, relapsed DLBCLs showed a lower number of chromosomal rearrangements when compared with the corresponding primary DLBCLs. Few dogs had the same profile, suggesting a linear clonal evolution; however, the majority of dogs showed a more complicated profile in terms of aberrations, and new rearrangements were also detected. Different scenarios can explain this molecular transformation at relapse. Chemotherapy alters the growth conditions of the neoplastic cells, thereby having a strong impact on the selection process of the resistant clones. If neoplastic cells dominating at diagnosis are sensitive to therapy, minor clones with intrinsic resistance may be secondarily generated by the tumor, and expand more efficiently once the competing clones are eliminated by the treatment. In dogs with the same pattern of chromosomal rearrangements, a reduced effect of the therapeutic protocol was confirmed. Our analysis identified relevant genes that may be also novel targets of chromosomal aberrations in relapsed DLBCL.

### Other low grade B-cell lymphomas

Although less frequently, transformation has been occasionally reported in other low grade B-cell lymphomas. Xing et al. (20) recently reported a transformation rate of 18% in splenic MZL. Meyer et al. (21) identified transformation, usually to DLBCL, as an independent unfavorable prognostic factor in MZL regardless of the origin (nodal, splenic, or MALT) and of subtype. The frequency of transformation of MZL is about 2.4% and is not far from that reported in FL (about 3%). Different mutations have been hypothesized to be associated with high grade transformation, but no definitive predictors of transformation have been recognized.

Canine MZLs are quite frequent in the spleen, mainly with a nodal architecture, often showing an indolent behavior, whereas nodal subtypes tend to exhibit a more aggressive behavior with a tendency to diffuse pattern and generalization (22). From a histological perspective, nodal MZL originates in a follicular fashion, but tends to progress in a diffuse fashion involving and compressing the entire lymph node. Valli et al. (22) have described this phenomenon as MZL late stage. Over 4 years, 26 dogs with nodal MZL were fully staged and treated at the authors’ institutions. Among them, one (3.8%) underwent transformation into DLBCL, which was histologically confirmed. Despite rescue treatment, the dog progressed and eventually died.

Transformation of lymphoplasmacytic lymphoma (23) has been rarely reported. MCL is generally aggressive, although rarely a more indolent behavior is reported. Transformation of MCL to aggressive pleomorphic MCL (24) or lymphoblastic leukemia (25) has been reported in humans.

In *dogs*, *MCL* is extremely rare (<2%) (18), and mainly confined to the spleen. Data on transformation are currently lacking.

According to the authors’ experience, canine B-cell small lymphocytic lymphoma (cB-SLL) can also undergo transformation. Among six cB-SLL diagnosed over 4 years, two (33.3%) underwent transformation into DLBCL, thereby representing the lymphoma histotype with the highest risk of histological transformation.

### T cell lymphomas

#### T-Cell Lymphomas

Although transformation has generally been focused in low grade B-cell lymphomas, several cases of transformed T-cell lymphomas are documented in human medicine.

Transformation of nodal T-cell lymphoma is rarely reported, since aggressive high grade lymphoma is highly prevalent in nodal forms.

In *dogs, indolent T*-*cell* lymphomas are not rare, mainly showing a T-zone derivation (TZL) (18, 26, 27). This subtype is characterized by long survival and indolent behavior. We recently described 51 TZLs (28). In those cases for which a complete follow-up was available, an indolent behavior was confirmed, and transformation never occurred. Interestingly, TZLs maintained a peculiar nodular architecture also in dogs having a clinical history of continuous relapse pattern and severe peripheral blood and bone marrow infiltration. A diffuse transformation seems not typical of this tumor.

On the other side, being one of the most diffuse indolent T-cell lymphoma in humans, MF (cutaneous epitheliotropic lymphoma) is frequently reported to progress and transform to more aggressive, large cells, and high grade neoplasms (29–31). Some prognostic indices, including immunophenotype, folliculotropism, and the extent of skin lesions, have been identified (30). Transformation has been reported to occur in 20–50% of patients with advanced MF (32) and is generally linked to a poor prognosis.

*Canine MF* generally harbors a poor prognosis in contrast with the human counterpart (32). Studies with large caseloads are lacking, but in none of the reported case series transformation to aggressive large cell lymphoma has been reported (33–35).

## Conclusion

Transformation of indolent lymphomas into more aggressive histological subtypes is still widely unexplored in veterinary oncology, although some evidences exist as a support. Oncologists and pathologists should carefully consider transformation as a possible scenario, mainly in the case of B-cell lymphoma/leukemia, similarly to what reported in human medicine. Nevertheless, some differences exist, being mainly related to the different incidence of some lymphoma subtypes. Efforts should be made to collect an adequate caseload of canine indolent lymphomas in order to define prevalence of transformation and to identify possible biomarkers useful for understanding pathogenesis and stratify patients in risk groups. Based on our personal experience and in agreement with the human literature, the grave prognostic significance of transformation is clear, as dogs in which it occurs had a very poor outlook, rendering transformation a therapeutic challenge.

## Conflict of Interest Statement

The authors declare that the research was conducted in the absence of any commercial or financial relationships that could be construed as a potential conflict of interest.
